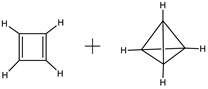# Information Entropy in Chemistry: An Overview

**DOI:** 10.3390/e23101240

**Published:** 2021-09-23

**Authors:** Denis Sh. Sabirov, Igor S. Shepelevich

**Affiliations:** 1 Laboratory of Mathematical Chemistry, Institute of Petrochemistry and Catalysis, Russian Academy of Sciences, 450075 Ufa, Russia; shepelevichis@mail.ru; 2 Bashkir State University, Bulletin of Bashkir University, 450076 Ufa, Russia

**Keywords:** information entropy, chemical structure, electronic structure, molecular complexity, molecular ensemble

## Abstract

Basic applications of the information entropy concept to chemical objects are reviewed. These applications deal with quantifying chemical and electronic structures of molecules, signal processing, structural studies on crystals, and molecular ensembles. Recent advances in the mentioned areas make information entropy a central concept in interdisciplinary studies on digitalizing chemical reactions, chemico-information synthesis, crystal engineering, as well as digitally rethinking basic notions of structural chemistry in terms of informatics.

## 1. Introduction

Information entropy (Shannon entropy) originates from the first quantitative theory of the communication and transmission of information [1,2]. It initially related to the complexity of a transmitted message [1] but now it has been adapted in diverse sciences [3]. In addition to the parent field, it is currently used to describe the objects of mathematics (e.g., graphs and sets), natural sciences (dissipative structures in physics, electron density, complexity of chemical and biological systems, etc.), engineering (urban infrastructure, analysis of images, etc.), and liberal arts (texts, etc.) [3,4]. This list is not complete and is permanently extending as information entropy is efficient for assessing the complexity of various objects.

‘Pure’ chemical applications of information entropy are wide and could be separated over the two major areas: (a) analysis of molecular graphs and (b) analysis of electron density of molecules. As follows from the names of the points, information entropy is mainly applied to the molecular species described with the finite mathematical models. The first group of the applications deals with the information entropy of molecular graphs that is very seminal for introducing various entropy-based topological descriptors for physical organic chemistry, digital chemistry, and QSAR/QSPR studies (quantitative structure–activity and structure–property relationships). These applications have been systematically reviewed in previous works [5,6,7,8,9]. The second group deals with the quantum-chemical analysis of the electron density distribution in the molecules and redistribution upon their chemical transformations (e.g., see [10,11,12,13]). We also mention in brief other chemical applications such as signal processing when molecules act as signal carriers (e.g., in the molecular switches based on the transits between the isomeric species) [14].

Despite the comprehensive reviews on chemical applications of information entropy, some recent advances deserve separate mentioning, especially in the context of existing theories and hypotheses, which are not familiar for broad chemical community. Additionally, some issues of information entropy permanently accompany relevant chemical studies, e.g., a comparison of information and thermodynamic entropies. Hence, it would be insightful to review the following points under one title:

(a) the peculiarities of the calculations of information entropies of isolated molecules, molecular ensembles, and solids;
(b) the relation of information entropies to chemical and physicochemical processes;
(c) the relation of information entropy to the digital recognition of chemical structures.

We consider that the solution of the interrelating problems from the above fields relate to deeper use of the concept of information entropy in chemistry, especially in the field of digital description of chemical processes.

## 2. Basic Definitions

The original Shannon’s approach to evaluating the information of a message treats it as the set of symbols: *x*_1_, *x*_2_, …, *x_n_*. The frequencies of their appearance are equivalent to the probabilities to find them in the message, *p*_1_, *p*_2_, …, *p_n_*. The information entropy (*h*) is introduced as [2]:

(1) $$h=-\sum_{i=1}^{n}p_{i}\log_{2}p_{i}v$$

(2) $$p_{i}=\frac{N_{i}}{N}$$

(3) $$\sum_{i=1}^{n}N_{i}=N$$

(4) $$\sum_{i=1}^{n}p_{i}$$

where *N_i_* are the numbers of symbols *x_i_* in the message, and *N* is its total length. The base of the logarithm in Equation (1) is arbitrary and usually it equals 2 or *e*, providing *h* estimates in bits or nats, respectively [15].

Shannon’s approach has been criticized due to its one-sided treatment of information. Indeed, it quantifies the messages in the context of the symbol’s appearance but ignores the sense of the message, i.e., the semantic aspects of information (in other words, the same set of the symbols is able to produce different messages) [16,17]. This feature prescribes the limits of the applications, including chemical ones, but does not make its use narrow.

The above formulation is also called the discrete information entropy approach and there is a continual analogue, as follows:

(5) $$h=-\int^{\text{​}}p\left(r\right)\ln p\left(r\right)dr$$

corresponding to the following continual probability distribution with random variable *r* [12]:

(6) $$\int^{\text{​}}p\left(r\right)dr=1$$

The proximity of Equations (1) and (5) providing *h* values to thermodynamic entropy (*S*) explains the use of the term ‘entropy’ for it. Thermodynamic entropy is introduced as:

(7) $$S=k_{B}\ln W$$

where *k*_B_ is Boltzmann’s constant. The *S* value describes a particular macrostate that is yielded from *W* number of microstates (various combinations of particles in various energy states). Herewith, the microstates are equally likely, so that the probability of a given microstate is *p_i_ =* 1/*W*. Thus, it is not surprising that the information and thermodynamic entropies are usually compared with focus on their similarity and linking theories and experiments are developed (e.g., Szilard engines and Landauer’s principle [18,19,20,21,22]). The interaction of information theory with thermodynamics and quantum theory has led to the generalization of the information entropy concept (cf.: Rényi and von Neumann entropies) [15,23,24]. However, mainly the quantities introduced with Equations (1) and (5) have taken root in chemical studies.

The way of using the discrete and continual information entropy approaches depends on the level of detailing chemical structure. This is due to the multilayered nature of chemical reality (term after G. Bachelard’s philosophizing on chemical sciences [25]). Indeed, there are many modes to describe molecular structures that take into account empirical formulas, molecular graphs, geometry, or conformational dynamics [26,27,28,29]. The required level of detailing structure is completely attributed to the aim of the chemical study. We discuss below three chemical applications, from most used to the least, with a focus on the molecular topology.

## 3. Information Entropy for Describing Chemical Structures

### 3.1. Discrete Information Entropy Approach: Quantifying Molecules as a Set

The common approach to rationalizing and quantifying molecular compounds deals with molecular topology [30,31]. This level of structural chemistry does not focus on electronic structure of atoms. A molecule is represented as a molecular graph **G**, which is a non-oriented colored graph (the coloring is necessary when the molecule is made up with the atoms of different chemical elements to designate the corresponding difference of the vertices of the molecular graph). The application of information entropy to analyzing the features of molecular graphs has a long story perfectly described in previous works [31,32]. We highlight below the conceptual aspects and some actual applications.

In general, this type of application deals with selecting and accounting inequivalent structural primitives (atoms, bonds, or molecular fragments) [5]. For this purpose, one should choose the equivalence criterion (α) and apply it to the set of the graph’s elements *X*. This produces a partitioning with respect to X into *n* subsets whose cardinalities are denoted by |*X_i_*|. The structural information content of a molecular graph is estimated similar to Equations (1)–(4) [32]:

(8) $$I\left(G,\alpha \right)=-\sum_{i=1}^{n}\frac{\left|X_{i}\right|}{\left|X\right|}\log_{2}\frac{\left|X_{i}\right|}{\left|X\right|}$$

Elements X classified with criterion α may be different. The development of the above approach went through the consideration of only empirical formula, empirical formula and atomic valences, diversity of the edges (chemical bonds) in the graph, its automorphic transformations, and the adjacency matrix (see review [31]). However, the use of Equation (8) most understandable by chemists is based on counting inequivalent graph vertices and graph edges corresponding to quintessential chemical concepts, atoms, and chemical bonds. Herewith, the application of Equation (8) to the vertices seems stricter as the atoms in the molecules are uniquely identified [33]. In contrast, a chemical bond is a vague concept because its strict criteria are absent [33], and when selecting chemical bonds in the molecule, chemists are guided by intuition (in most cases, such an ‘intuitive approach ’ works well but there are debatable examples, especially related to coordination compounds, endohedral complexes, molecules with multicenter chemical bonds [33,34,35,36]). Note that Bader’s theory Atoms-in-Molecules, whereby the concept of chemical bond is replaced with ‘chemical bonding’, efficiently resolves some structural problems [37]. However, it also leads to disputable results for ‘no-doubts’ molecular systems (e.g., it indicates H…H bonding in phenanthrene bays of polycyclic aromatic hydrocarbons, hardly interpretable by classical chemical theory [38,39]). Nevertheless, in simple molecules with undoubted structural formulas (e.g., fullerenes or hydrocarbons), the edge-based approach works reliably.

**Figure 1:**
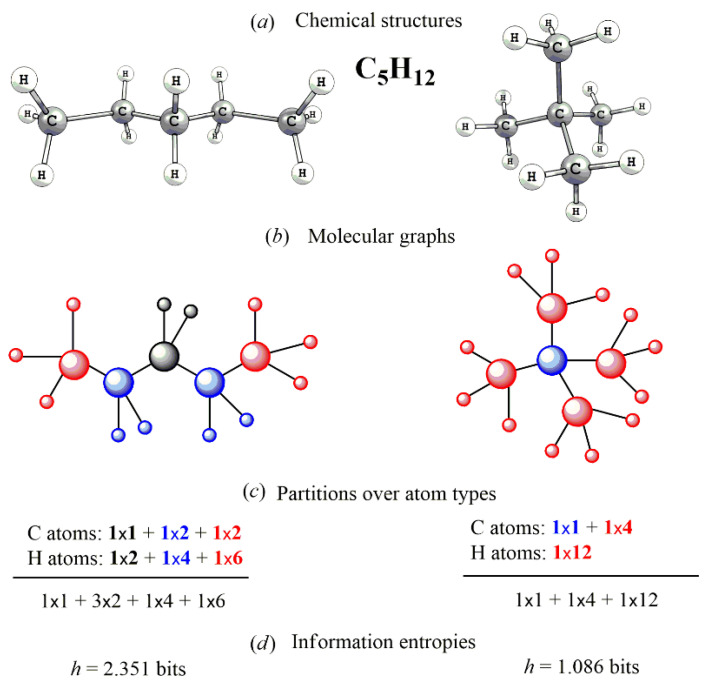
Explanatory scheme of calculating the information entropies of two C_5_H_12_ isomers, pentane (left) and neopentane (right). Taken from [47]. The molecules (**a**) are digitalized as graphs (**b**) whereby the inequivalent vertices are selected. Then, the counting the atom types and their populations (**c**) are performed and information entropies are calculated via Equation (9) (**d**) © 2020 Elsevier.

Equation (8) applied to graph vertices was widely used by Bonchev’s [40,41], Basak’s [42,43], and our [44,45] groups. In these works, the following values,

(9) $$\tilde{I}=-\sum_{i=1}^{n}\frac{N_{i}}{N}\log_{2}\frac{N_{i}}{N}=\log_{2}N-\sum_{i=1}^{n}\frac{N_{i}}{N}\log_{2}N_{i}=h$$

and

(10) $$I=N\tilde{I}=N\log_{2}N-\sum_{i=1}^{n}N_{i}\log_{2}N_{i}$$

are called information content (and designated as IC or *h*) and the total information content of the molecules, respectively. The units of these quantities are bits/atom and bits, respectively. The mentioned works exploit several modes of partitioning the vertices: (i) chromatic-number coloring of **G** where two vertices of the same color are considered equivalent; (ii) determination of the orbits of the automorphism group of **G** whereafter vertices belonging to the same orbit are considered equivalent. To provide reliable results, sortation of the atoms have to reflect their chemical inequivalence regardless of the used mathematical protocol. It means that atoms of different elements are attributed to different atom types (herewith, atoms of different elements equally contribute into the information entropy of the molecule); atoms of the same element belong to different atom types (subsets) if they occupy different positions in the graph. The inequivalence of the vertices depends on the connectivity, which, however, is not explicitly considered, i.e., the types of the bonds (single, double, triple, or coordination bond) do not matter [46]. The approach is exemplified with the case of two isomeric hydrocarbons C_5_H_12_ (Figure 1; conventional designation to describe the partition of the molecule: [number of atom types] × [number of atoms within them] is used hereinafter). The calculated *h* values of all possible *N*-atomic molecules with *N* = 2–4 (Table 1) and more complex species (Table 2) are presented.

**Figure 2:**
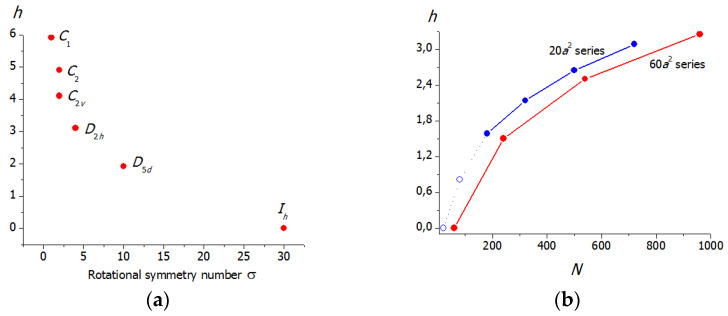
Relations of the information entropy of fullerenes to the molecular structure and molecular size: (**a**) information entropy vs. rotational symmetry number plot for the C_60_ isomers; and (**b**) dependence of information entropy on the number of atoms of the molecules of icosahedral fullerenes from two Goldberg series. Taken from [44] © 2015 American Chemical Society.

**Figure 3:**
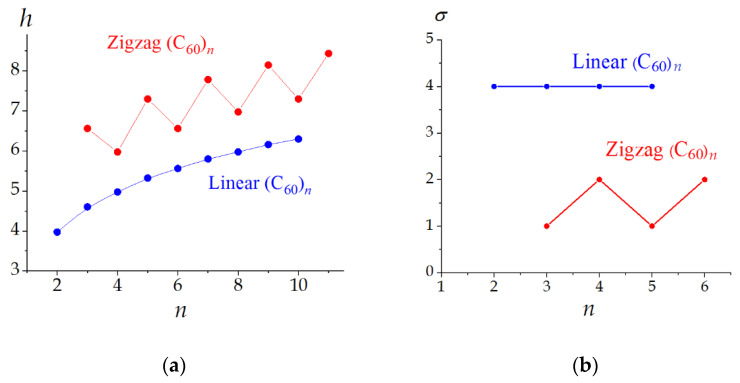
Relations of the information entropy (**a**) and rotational symmetry number (**b**) for fullerene oligomers. Taken from [49].

The application of Equation (9) to various molecules and hypothetical molecular graphs showed that information entropy characterizes the structural (topological) complexity of the molecule and depends on its elemental diversity, the number of the constituting atoms (factors increasing *h*), and the symmetry (reduces resulting *h*). These factors can be clearly exemplified with the rigid molecules, e.g., fullerenes. For example, in the set of the isomeric C_60_ molecules with different symmetries (Figure 2a), the *h* values decrease with the rotational symmetry number σ. However, different symmetries lead to the same *h* values if they relate to the same partitions (e.g., the molecules consisting of *N* atoms and having *C_i_* and C_2_ symmetries have the same partitions, (*N*/2) × 2, and the same *h* values, equal to $-\log_{2}\frac{2}{N}$). In the case of the fixed symmetry, the information entropy regularly increases with the molecular size (Figure 2b) [44]. Information entropy reflects the symmetry alternations in homological series. For example, the symmetry oscillates in the series of zigzag oligomers (C_60_)*_n_*, being *C*_2*h*_ for all even and *C_S_* for all odd homologues. Accordingly, the dependence of *h* on *n* has a saw-like view (Figure 3). In contrast, all linear structures (C_60_)*_n_* have *D*_2*h*_ symmetry, and function *h* = *f*(*n*) monotonously increases [48,49].

**Figure 4:**
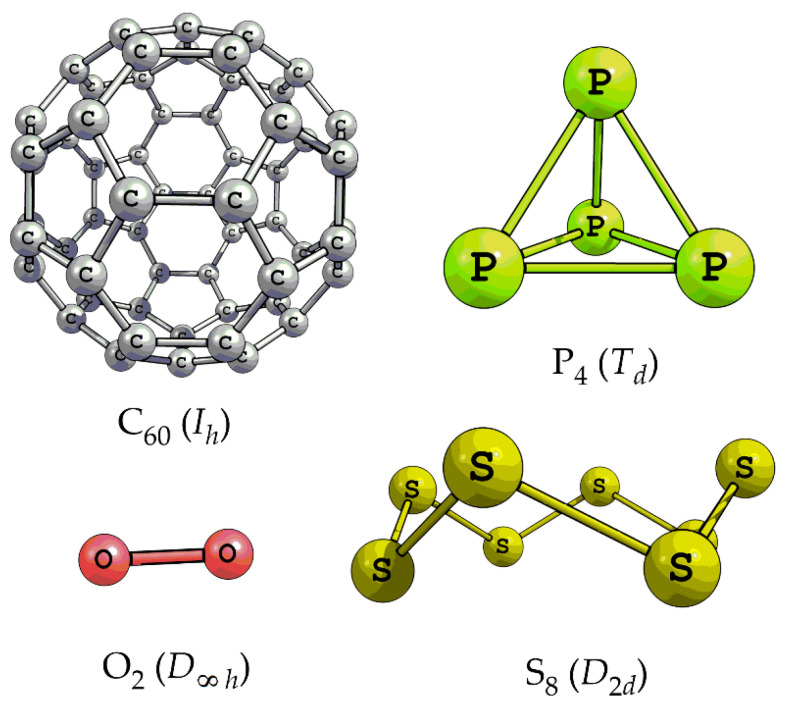
Typical molecular species with zero information entropy. Taken from [50].

The limit values of function *h* = *f*(*N*), where *N* is the number of atoms in the molecule, are worth mentioning. Symmetric molecules with uniform structures, i.e., with partitions 1 × *N*, have zero information entropy. These are all homonuclear diatomic molecules and rare symmetric species with larger molecular size (Figure 4). Their antipodes with partitions *N* × 1 are non-symmetric and have the maximal *h* value for a given *N*:

(11) $$h_{max}=\log_{2}N$$

This approach allows sorting fullerenes [44], endofullerenes [51], oxygen allotropes [45], and interstellar molecules [52] to reveal more and less abundant structures. We performed studies [44,45,51] hypothesizing that a lower possibility of the formation of a chemical structure corresponds to the higher information in the “message” about its formation. The equivalence of the atom types implies the possibility of the formation of stable structures. We assumed that the more complex the structure, the lesser the probability of its formation. Such probabilistic nature of forming chemical structures regardless of the stability corresponds to the non-equilibrium and extreme conditions [53].

**Figure 5:**
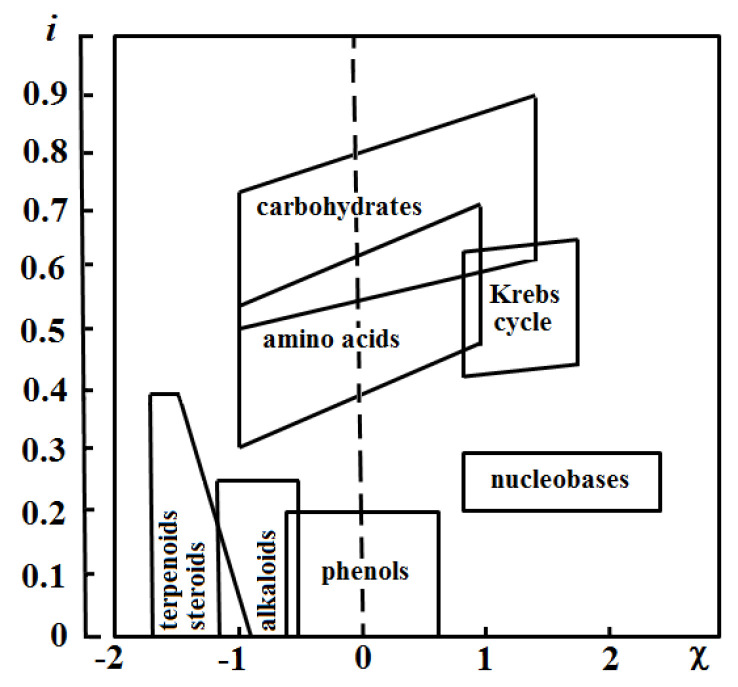
Specific information entropy (*i* = *I*/*N*) vs. formal oxidation state (χ) of carbon atoms of natural compounds. Taken from [57].

The approach provides additional opportunities for classifying chemical structures. It could be done with the use of *h*-based estimates in combination with other parameters relevant to chemical properties of the substances [54,55,56,57]. For example, Zhdanov proposed the classification of natural compounds in the space of two coordinates: *h*/*N* and the mean oxidation state of the carbon atoms in the molecule (Figure 5) [57]. The *h* values were efficient for the rationalization of the formation processes of singly and doubly filled endofullerenes [51]. However, this approach itself was not helpful for understanding addition reactions to fullerenes [58] and ‘energy–topology’ correlations in isomeric fullerene series [59]. Hence, the idea of the use of *h* in its original form has limited applicability to assessing the reactivity and stability of the molecules.

Another classification opportunity following from the discrete information entropy approach exploits the concept of isentropicity. Two molecules A and B are isentropic if their information entropies are equal (*N*_A_ and *N*_B_ are the total numbers of atoms in the molecules) [50]:

(12) $$\sum_{j=1}^{n_{A}}\frac{N_{Aj}}{N_{A}}\log_{2}\frac{N_{Aj}}{N_{A}}=\sum_{i=1}^{n_{B}}\frac{N_{Bi}}{N_{B}}\log_{2}\frac{N_{Bj}}{N_{B}}$$

**Figure 6:**
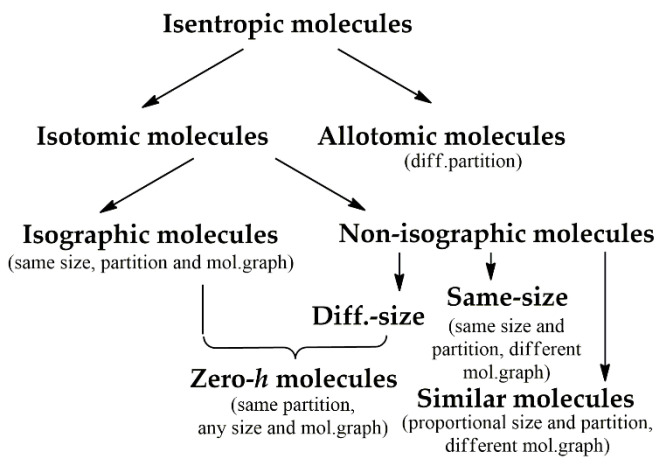
Classification of isentropic molecules with the distinctive properties of their structures. Taken from [50].

There is no overall solution of Equation (12), as parameters *N_i_*, *N_j_*, *N*_A_, *N*_B_, *n*, and *m* may be varied. Additionally, the partition of each molecule and the corresponding cardinalities of the subsets (*N_i_* or *N_j_*) are generally interdependent. However, particular cases of the above parameters when fulfilling the condition of isentropicity have been considered. We proposed the *h*-based classification tree of the molecules (Figure 6) that includes isotomic (having the same partition and size or similar partition and different sizes) and allotomic (differently partitioned) [50]. Using the tree, we remember a disadvantage of the Shannon approach dealing with neglecting the semantic properties, and it must be taken into account when using such classifications. Hence, the question how to distinguish cases when isentropicity has structural/physical meaning and when it has not remains open.

Basak et al. introduced several related indices. Structural information content (SIC) and complimentary information content (CIC) are the most important among them:

(13) $$\mathrm{SIC}=\frac{h}{h_{max}}$$

(14) $$\mathrm{CIC}=h_{max}-h$$

The structural information content obtains the values from 0 to 1 (or from 0 to 100%) and shows the degree of the realized complexity relative to the maximal complexity achievable for the same number of atoms *N*. The use of SIC values allows for comparing the complexities of the molecules with different sizes. The molecules with the maximal *h* values are characterized with SIC = 1. We have used this descriptor for analyzing the set of interstellar molecules and found that the most hydrogen-poor unsaturated molecules have rigid structures and SIC = 1. In contrast, the hydrogen-rich interstellar molecules have SIC < 1 [52].

Chemical bonds of the molecules (or edges of the corresponding molecular graphs) can also be used as structural primitives when calculating with Equation (8) [60]. For example, Basak et al. introduce bond information content similar to the SIC value [5,43]. Different entropy measures relating to the topological distances in the fullerene [61,62,63,64] and dendrimer graphs [65] also demonstrate usefulness.

**Figure 7:**
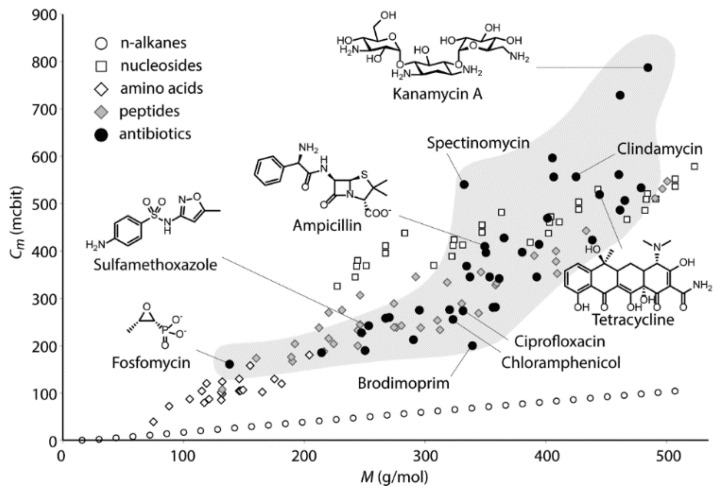
Molecular complexity vs. molar mass of selected organic molecules. Taken from [68] © 2016 American Chemical Society.

Efficient logarithmic measures of molecular complexity uniting atom and bond approaches are also known [66,67,68,69]. For example, Böttcher has developed index *C_m_*, which digitalizes molecular complexity in more details as compared with original Shannon’s formula [68], as follows:

(15) $$C_{m}=\underset{i}{\sum }d_{i}e_{i}s_{i}\log_{2}V_{i}b_{i}-\frac{1}{2}\underset{j}{\sum }d_{j}e_{j}s_{j}\log_{2}V_{j}b_{j}$$

where $\log_{2}V_{i}b_{i}$ represents the basal terms of the equation deduced for *i*-th atom from the number of valence electrons (*V_i_*) and the number of bonds connecting this atoms with its neighbors (*b_i_*); *d_i_* is the number of inequivalent bonds to neighboring non-hydrogen atoms (having *V_i_b_i_* > 1); *e_i_* is the heteroatom diversity parameter; and *s_i_* is the number of isomeric possibilities at the *i*-th position. In Equation (15), to account for the symmetries of a molecule, the corresponding atom positions of chemically equivalent sets of atoms for each symmetric position *j* are subtracted. This approach provides efficient assessing the complexity of chemical structures (Figure 7). However, grown from the information theory, index *C_m_* mathematically seems to partly lose the probabilistic nature of the original Shannon entropy.

It is noteworthy that there is no universal conception of chemical complexity [70], so that the information entropy estimates is just an approach among the others. In benchmarking work [6], Bonchev has compared three approaches to assessing chemical complexity, algorithmic, information-theoretic, and topological, and demonstrated that they can lead to qualitatively different estimates. For example, highly symmetric molecules obtain low *h* values, i.e., they have a uniform distribution of atoms over atom types, and we consider them simple within the information-theoretic approach. The situation is completely different when the algorithmic complexity is used. In this case, the symmetry elements intrinsic to a symmetric molecule are described with a digital protocol. The higher the symmetry point group, the larger the protocol and the higher the molecular complexity. Hence, symmetric molecules are complex in terms of the algorithmic approach.

We briefly note at the end of this section that the concept of information entropy has been generalized, giving birth to numerous entropy-based descriptors [23,24,32].

### 3.2. Continual Information Entropy Approach: Quantifying Electronic Density of Atoms and Molecules

In quantum chemistry, the Hartree–Fock and Kohn–Sham [71] methods claim a simple relation for one electron between its wave function *ψ* and electron density *ρ*:

(16) $$\rho_{i}\left(r\right)={\left|\psi_{i}\left(r\right)\right|}^{2}$$

The electron density is the measure of the probability of an electron being present at an infinitesimal element of space surrounding any given point. As it has a probabilistic origin, it could be substituted into Equation (5), representing infinite cases of the information entropy. Guiding these considerations, the *N*-electron system has the information entropy equal to [4]:

(17) $$S_{\rho }=-\int^{\text{​}}\rho \left(r\right)\ln\rho \left(r\right)dr$$

(18) $$\int^{\text{​}}\rho \left(r\right)dr=N$$

Another quantity *S*_σ_ is also used within this approach. It is obtained when using in Equation (17) a normalized function *σ*(**r**) = *ρ*(**r**)/*N* called the shape factor [4,72,73]:

(19) $$S_{\sigma }=-\int^{\text{​}}\sigma \left(r\right)\ln\sigma \left(r\right)dr$$

(20) $$\int^{\text{​}}\sigma \left(r\right)dr=1$$

Equations (17) and (19) have been applied to analyze the electron density of atoms, molecules, and their parts (chemical bonds or fragments). The physical sense of the *S_ρ_* and *S_σ_* values relates to the degree of delocalization of electron density [72,74,75].

An interesting approach based on Equation (17) was introduced to quantify the aromaticity of organic compounds [75]. The logic of the approach is based on Bader’s theory of Atoms-in-Molecules [37], and the starting parameters are the electron densities in the bond critical points (BCPs) that correspond to intramolecular interactions, including chemical bonds. To assess the aromaticity of the ring, the local information entropies in each BCP of the aromatic cycle are calculated as follows:

(21) $$S_{i}=-p_{i}\ln p_{i}$$

(22) $$p_{i}=\frac{\rho_{\mathrm{BCP},i}}{\sum_{i=1}^{N}\rho_{\mathrm{BCP},i}}$$

The total information entropy of the system of chemical bonds comes from the summation over all BCPs:

(23) $$S_{\rho ,tot}=\sum_{i=1}^{N}S_{\mathrm{BCP},i}$$

All BCPs are the same in the idealized aromatic system with total delocalization, so that *p_i_* = 1/*N* and the total information aromaticity equals:

(24) $$S_{\rho ,max}=\ln N$$

Deviation of the information entropy of a studied system from this maximal value could be used as a measure of aromaticity, and the authors of [75] call it Shannon aromaticity (SA):

(25) $$\mathrm{SA}=S_{\rho ,max}-S_{\rho ,tot}$$

The approach seems informative, and SA values correctly describe known aromatic systems. For example, SA ≈ 0 in the case of benzene, the golden standard of aromaticity. The values obtained for bi- and tricyclic aromatic hydrocarbons are shown in Table 3.

As for the application of the continual information entropy approach to chemical reactions, we pay attention to two works [73,76]. The work of Ho et al. [76] used *S_ρ_* and some related quantities to check their changes upon simplest S*_N_*2 displacement reactions. Although function *S_ρ_* = *f*(ξ) (ξ is the reaction coordinate) still shows the redistribution of electron density upon the reactions, the authors have not found any specific behavior of the function in the stationary points lying on the reaction path (minima of reactants/products and saddle point of a transition state).

Geerlings and Borgoo [73] were more successful. They did not use information entropy itself but the derived quantity, viz., the Kullback–Leiber information deficiency along the reaction coordinate relative to the reference value corresponding to the transition state (TS):

(26) $$\Delta S_{\mathrm{KL}}=\int^{\text{​}}\sigma_{\xi }\left(r\right)\ln\frac{\sigma_{\xi }\left(r\right)}{\sigma_{\mathrm{TS}}\left(r\right)}dr$$

**Figure 8:**
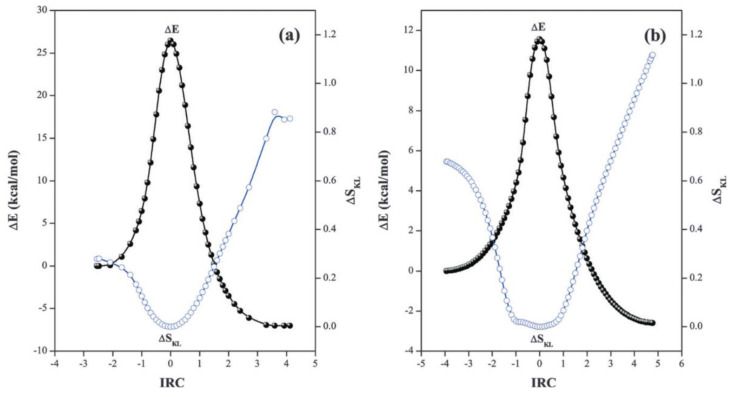
Energy and global Δ*S*_KL_ information deficiencies along the intrinsic reaction coordinate (IRC) for (**a**) intramolecular (HO–N=S → O=N–SH) and (**b**) intermolecular reactions (HO–N=S…OH_2_ → O=N–SH…OH_2_). Taken from [77] © 2009 Royal Chemical Society.

Using Equation (26), the authors of [77] found that the minimum of Δ*S*_KL_ corresponds to the activation barrier and TS of the studied chemical reactions (proton transfer and S_N_2 reaction) (Figure 8).

A deep systematic study was performed by Nalewajski [10,11] combined in his studies the above mathematical apparatus with basic definitions of the density functional and Atoms-in-Molecules theories. One of the advances of these works deals with the application of the continual information entropy approach to communicating (interacting) molecules, i.e., molecules in a pre-reactionary ensemble [10,11,78,79].

There are a lot of works on chemical applications of the continual information entropy approach. However, its discrete counterpart seems more efficient and more widespread in chemistry. Indeed, the information-entropy-based electronic structure parameters are deduced from wave functions, which are the base for calculating numerous other quantities and quantum-chemical descriptors [71]. These quantum-chemically calculated values (energies of frontier molecular orbitals, dipole moments, polarizabilities, hardness, etc.) easily replace information-entropy-based ones. Herewith, in most cases, the traditional indices of electronic structure and reactivity are more justified. Another issue deals with the used quantum-chemical approximations to calculating the parameters presented in this section as they strongly depend on the quality of a quantum-chemical method.

Note that there are minor examples of chemical applications of the continual approach, which do not deal with electron density. As an example, we mention here a methodology for assessing configurational entropy of macromolecules [80].

### 3.3. Chemical Applications of Information Entropy Relating to Signal Processing

As is known, molecules act as information carriers and could be involved in the information processes [81,82,83]. They are able to be the source of a signal as well as the convertors. In the first case, a whole molecule or its distinguishable parts (monomer units of biopolymers, e.g., amino acid or nucleotide residues). For information recording, the molecular systems with two (or more) stable states are very significant if the stable have different measurable spectroscopic parameters. Additionally, the transits between the states should be only due to a specific impact, e.g., irradiation with light with a specific wavelength. The uncertainty arises even in the simplest case of acting bistable system A ↔ B. In a kinetic aspect, when system moves from one state to another, states A anb B coexist and their ratio is changed in time. The coexisting affects signal processing. In the equilibrium thermodynamics, the population of *i*-th state (isomer) of such a system can be found as:

(27) $$q_{i}=\frac{\exp\left(-\frac{\Delta E_{i}}{RT}\right)}{\sum_{i=1}^{n}\exp\left(-\frac{\Delta E_{i}}{RT}\right)}$$

where Δ*E_i_* = *E_i_* − *E*_0_ are the stabilities of the states (isomers) relative to the most stable one *E*_0_. Obviously, the *q_i_* values can be interpreted as probabilities, and they meet criterion $\sum_{i=1}^{n}q_{i}=1$. Therefore, they could be used as the probabilities for Equation (1) and input parameters of the information entropy approach. Notably, these values are close to physical interpretation of the measurements of the systems existing as mixtures. For example, anisotropy of polarizability [84] and refractivity index of such systems of mixed states [85] have been estimated as below, and we think this mode is applicable to any scalar physical quantity *x*:

(28) $$x_{system}=\sum_{i=1}^{n}q_{i}x_{i}$$

**Figure 9:**
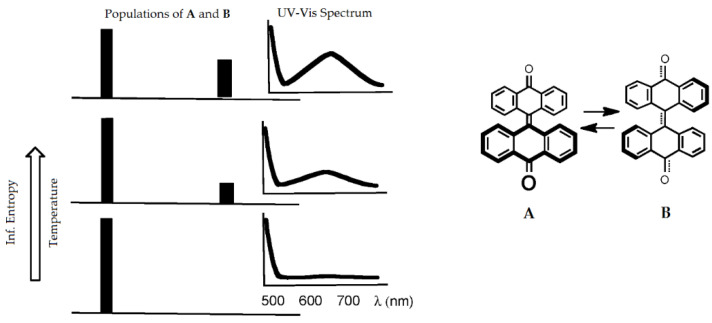
Information in the bistable thermochromic system of two interconverting bianthrone isomers implies ‘a chemical message’ associated with color/spectral changes [14].

The above considerations were successfully applied to the analysis of dynamics of multi-stable systems, such as the mixture of two photochemically interconverted isomers [14] (Figure 9). It is also applicable to more complex processes involving enzymes [86]. Thus, the uncertainty of *x_system_* highlighted with information entropy *h*(*q_i_*) could be closer to the measurable physicochemical parameters of the system.

Information entropy is also used for rationalizing surface processes: e.g., in the analysis of the scanning tunneling microscopy images of epitaxial fullerene nano-aggregates [87] or tribological processes [88]. A recent machine learning study of digitalizing surface processes [89] demonstrated that the surface is describable in terms of the set theory, for which information theory is more than suitable.

## 4. Information Entropy of Complex Chemical Objects

### 4.1. Information Entropy of Solids

Representing an isolated molecule as a set is accompanied with no questions: the representation is unambiguous even if arranging chemical bonds is unclear. Trying to consider a crystalline solid, we must take into account the inequivalence of its layers (e.g., the presence of internal and surface parts) and defects (vacancies or inclusions). Considering the solid as a whole in the same manner as a molecule lead to the conclusion about the inequivalence of all atom positions, so its information entropy is assessed with Equation (11). Tatevsky declares that it is reasonable to consider the solid macroscopic if it contains more than 10^4^–10^8^ atomic nuclei [90] that corresponds to *h* = 13.29–26.58 bits/atom. Thus, the information entropy of the solids in line with these considerations depends only on their size. These considerations seem precise but uninformative, as they do not allow comparing the information entropies in the context of chemical structure. Thus, it should be modified to be suitable for chemical studies. If we deal with a crystal, we should treat it as infinite structure with no defects to escape the mentioned inequivalence.

Krivovichev systematically develops the information entropy approach applied to crystals [91]. Accordingly, the measure of the information entropy of a crystal relates to the information entropy of the reduced unit cell. All cells are considered identical. The main quantities of the approach are structural information content and total information content calculated with Equations (9) and (10) and expressed in bits/atom and bits/unit cell, respectively. The inequivalence of the atom’s positions within the cell is considered similar to the case of the isolated molecules. Additionally, parameter *d* referred to the volume of the reduced cell (*V*) is efficient for crystal entropy studies:

(29) $$d_{inf}=\frac{I}{V}$$

It is called information density and expressed in bits/Å^3^. Krivovichev with collaborators and followers applied these quantities to study various minerals [91,92,93,94,95,96,97,98,99,100,101] and found the relations between the crystallographic symmetries, size, and information entropy estimates (Table 4) and proposed the classification of the minerals, which is based on their complexity estimated with *I* values (Table 5). The latter means that the information entropy is associated with the complexity of the minerals (though the authors make a reservation that other approaches to assessing the complexity of the minerals can be used, such as exploiting the approximation of the crystal structures as the nets or invoking algorithmic complexity paradigm [96]). As reported, the following ranges are observed in the case of the five most complex minerals: $\tilde{I}$ = 5.730…8.622 bits/atom, I = 6111….23,477 bits/cell, and *d_inf_* = 0.488…0.791 bits/Å^3^ [93].

Introduced as above, the information entropy of minerals ($\tilde{I}$) is relevant to the configurational entropy of the solid *S_config_* [95]:

(30) $$S_{config}^{max}-S_{config}=k_{B}N\tilde{I}\ln2$$

where *N* is the number of atoms in a crystal and ln 2 is hereinafter the conversion factor between the natural and binary logarithms of the left and right parts of Equation (30). As follows from the equation, configuration entropy reduces the structural complexity of a crystal [95].

**Figure 10:**
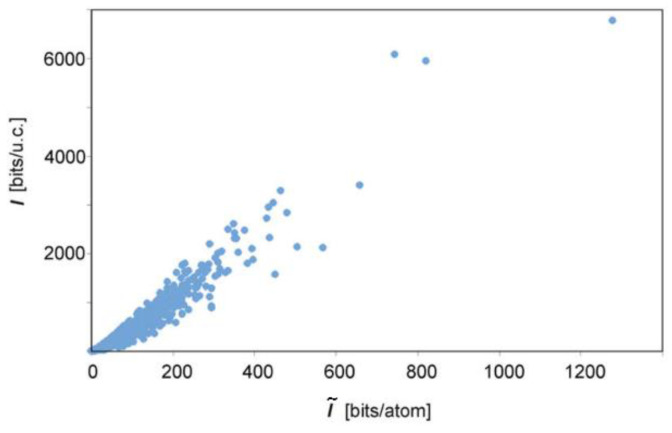
The total information content of minerals plotted against the information content per atom. The plot is based on data from the works of Krivovichev group [91,96].

Analysis of a large data on crystalline structure minerals using the above approach allowed revealing some general correlations typical for the mineral world, e.g., the correlation between I and $\tilde{I}$ (Figure 10). For selected classes of minerals, both $\tilde{I}$ and I values reflect structural complexity and correlate with chemical complexities estimated as the information entropies of chemical composition (when the contents of the unit cell or empirical formula are used as the input data for Equations (9) and (10)) [94,99].

The information entropy values digitalize the fact that the structures of the molecules in the isolated state may not correspond to their structures in the crystals [100]. Their original symmetries are reduced upon forming molecular crystals due to the influence of the neighbors of a crystal lattice (Table 6). Note that additional factors in addition to the structural complexity (e.g., layer stacking or complexity of hydrogen network) can be taken into account within the approach described above [97].

As the structural complexity of the minerals is relevant to their physicochemical properties, the correlations between the information entropy and energy parameters of the minerals are being discovered. For example, the structural complexity of lead-containing minerals may have an impact on mineral reactions when the system does not have the energy sufficient for overcoming the energy barrier separating metastable simple structures from stable complex ones [91]. The correlation between the total information content of the Cu_2_(OH)_3_Cl polymorphs and their Gibbs formation energies have been reported [101].

Dendritic structures (fractals) were presented as the graphs, whose vertices correspond to elementary units (the inner structure of the units was not considered) [102]. As found, the information entropy approaches to the threshold values with the increasing generation number and these limited information entropies depend on the branching parameter of the structure.

Describing the information entropy of condensed matter relates to copying the polyatomic systems. Herewith, it is not a problem to create a copy of the molecule. However, the production of exact copies of chemical structures with number of atoms *M* >> 10^4^ by means of traditional physical and chemical techniques is a challenge. For example, glasses with the same chemical composition produced with identical synthetic protocols have almost the same macroscopic properties, but they are not the copies [103]. This inconsistency is not decisive for most macroscopic solids but becomes crucial for nanostructures because the variations of their structures even in a narrow range could induce drastic changes in the properties. This challenge links to the physical task of defining maximal information *I* (in bits), which could be recorded and stored for a long time *t_max_* with the system of *M* atoms. This question was systematically studied by Bal’makov [103,104,105,106,107], who proposed the following inequality for this purpose:

(31) $$0\leq I\leq \frac{M\beta \left(t_{max};n\right)}{\ln2}$$

where *β* (in nats) is the specific information capacity depending on *t_max_* and vector **n** of relative concentrations of atoms attributing to different types. Note that *I* designates the information in a macroscopic sense and could vary with recording and storage techniques, whereas *β* is independent of the latter features and is defined by microscopic parameters. The exact estimate of *I* is vague but should be relevant to the number Γ of possible ‘recordable’ macro-states of the system (i.e., the sets of the atomic nuclei). This is due to the interpretation of recording information as the embodiment of one of Γ states. Thus, the upper bound of *I* should be lnΓ/ln2 [103]. Notably, Γ could be greater than the number of minima of the Gibbs energy of the considered polyatomic ensemble, which means the possibility of implementing the information into a metastable state.

Configuration entropy in terms of the above approach characterizes the scatter of the experimental results on determining the coordinates of the atomic nuclei of the system rather than the degree of disorder of a particular structure [104]. Anyway, the information entropy insights are one of the steps toward rationalizing the replication processes of artificial chemical structures and could be useful for developing approaches to ‘chemico-information synthesis’ [104,105,108,109,110].

The concept of chemico-information synthesis has been introduced by Aleskovsky only in a very general manner [108,111]. He starts hypothesizing with the absence of the solids and high-molecular organic compounds with reproducible chemical structure (vide supra), except of biopolymers. The latter ones exist due to other laws regulating their formation, which is based on the high complementarity of the reactants, their responses to weak impacts from the environment, etc. Aleskovsky unites all these factors under the concept of information and states that the structuring is the process of embodiment of information into matter, so that structure and matter are considered almost as synonyms. Biosynthesis involves the molecules with high information content. Therefore, the synthesis of their artificial inorganic analogs should be based on this feature and use the reactants with propensities for self-assembly or selective reaction under weak impacts. Metaphorically, the information is printed in the chemical structure. Its chemical transformation is associated with reading the information implemented in the structure of the reactants. Chemico-information synthesis must operate with the building blocks with high information contents instead of low-information ones (mainly atoms).

Talanov and Ivanov develop this idea refining the type of the structuring processes: iteration, dissymmetrization, modularization, and hierarchization [109]. Works [108,109,111] have proposed information entropy for assessing the information contents of the reactants, but their use has not been exemplified. As assumed in [109], the lower the probability of the formation of a certain structure under a stochastic process, the greater the information in the ‘message’ of its formation (i.e., the information entropy). Currently, these ideas are exteriorized in the studies on chemical reactions under weak physical fields (when the impact is less than *kT*) and the self-governing synthesis of nanomaterials (which implies the absence of any external impact on the synthesis since reaching the technological regime). Our group uses information entropy values for sorting most probable structures formed under non-equilibrium conditions [44,51,52] and ultrasonication [58].

### 4.2. Information Entropy of Molecular Ensembles

The discrete information entropy approach is promising for the description of collectives of molecules. To develop the corresponding computational technique, the concept of the molecular ensemble [112] of *m* molecules with information entropies *h_i_* was used and it was found [46,47] that

(32) $$h_{\mathrm{ME}}=H_{\Omega }+\sum_{i=1}^{m}\omega_{i}h_{i}$$

where *ω_i_* are the fractions of the molecules in the ensemble:

(33) $$\omega_{i}=\frac{N_{i}}{\sum_{i=1}^{m}N_{i}}$$

and the first term *H*_Ω_ is called cooperative entropy:

(34) $$H_{\Omega }=-\sum_{i=1}^{m}\omega_{i}\log_{2}\omega_{i}$$

As follows from Equation (31), the resulting information entropy *h*_ME_ of the ensemble is not a sum of the constituting molecules *h_i_*. This statement was mathematically derived and justified with the examples demonstrating the consistence of Equation (32) with chemical intuition [46,47].

We focus on the specific additive rules of information entropy of molecular ensembles because simple summation of *h* values of the molecules leads to counterintuitive results. For example, their simple summation means that in the case of the ensemble of *m* identical molecules, the information entropy reflecting the complexity of the ensemble increases linearly with *m*. This contradicts general chemical notions and thinking nuclear magnetic resonance experiments [46]. Equation (32) resolves the contradiction and the information entropy of the ensemble made up with the same molecules does not depend on their number (*h*_ME_ = *h*). The second illustrative example deals with the ensemble consisting of two molecules with *h*_1_ = 0 and *h*_2_ ≠ 0: the simple summation underestimates the information entropy, ignoring the increase in the ensemble’s complexity associated with zero-*h* molecule. This ‘missed’ contribution is accounted for with the *H*_Ω_ term.

**Figure 11:**
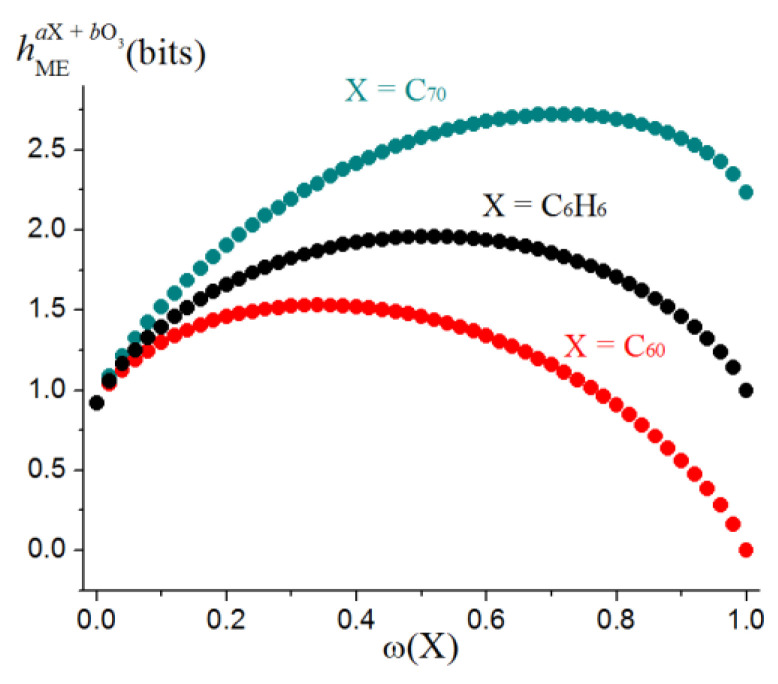
Information entropies of dimorphic molecular ensembles consisting of a carbon-based substrate and ozone *a*X + *b*O_3_ as a function of the fraction of the X molecules, ω(X). Taken from [47] © 2020 Elsevier.

The *h*_ME_ values for some typical dimorphic molecule ensembles (i.e., made up with the molecules of two types) and a general view of functions (32) are shown in Table 7 and Figure 11. Cooperative entropy *H*_Ω_ depends only on the distribution of atoms over the molecules of the ensemble. It does not relate to their structure and emerges because of mixing. The case of molecular ensembles is important for obtaining correct estimates of information entropy changes in chemical processes.

We have found ‘magic’ molecular ensembles with featured information entropy [47]. The ensemble of this type consists of *m* isentropic molecules (with entropies equal to *h*) and has the structure resembling the structure of the constituting molecules. Resembling means that the distribution of the atoms over the molecules in the ensemble is proportional to the distribution of the atoms over the atom types in the molecules. Such ensembles have the following:

(35) $$H_{\Omega }=h,\;\mathrm{and}\;h_{\mathrm{ME}}=2h$$

It means that the information entropy of such ensembles is independent of the number of constituting molecules and is defined only by their information entropies.

## 5. Information Entropy of Chemical Reactions

It is well known that the changes in thermodynamic functions are calculated as the differences between final and initial values. Applied to chemical reactions, it means that the change in function ΔΥ_R_ is the difference between the sums of the Υ values, products, and reactants:

(36) $${\Delta \Upsilon }_{R}=\sum_{prod}\Upsilon_{i}-\sum_{reat}\Upsilon_{j}$$

Guided by this analogy, Karreman [113] introduced the change in topological content at the chemical reaction estimated with *h* values of participants. Currently, the change calculated in line with [113] is called the structure-dependent reorganization entropy of chemical reaction:

(37) $$H_{reorg}^{str}=\sum_{i}^{prod}h_{i}-\sum_{j}^{react}h_{j}$$

The sense of Equation (37) is discussed below, and here we note that $H_{reorg}^{str}$ is insufficient to describe the information entropy change in chemical reaction (or simply information entropy of chemical reaction). According to the previous section, this change must be introduced as the difference between the information entropies of the ensembles of products and reactants:

(38) $$\Delta h_{R}=h_{\mathrm{ME}}^{prod}-h_{\mathrm{ME}}^{react}$$

The combination of Equations (32) and (38) after some regrouping allows the following equation for Δ*h*_R_ [47]:

(39) $$\Delta h_{R}=H_{redisrt}^{size}+H_{reorg}^{str}+H_{reorg}^{str+size}$$

where the first term is the entropy of redistribution of the atoms over the molecules upon chemical reaction. It comes from the difference between the cooperative entropies of the molecular ensembles:

(40) $$H_{redisrt}^{size}=H_{\Omega }^{prod}-H_{\Omega }^{react}$$

The second term is the information entropy change as introduced by Karreman [113]. It depends only on the structures of the molecules, formed and destroyed, and does not depend on their sizes. As the value defined with Equation (37) is associated with the intrinsic molecular changes, we called it a part of the reorganization entropy. Its other part, the third term of sum in Equation (39), depends on both molecular structure and size:

(41) $$H_{reorg}^{str+size}=\sum_{j}^{react}\left(1-\omega_{j}\right)h_{j}-\sum_{i}^{prod}\left(1-\omega_{i}\right)h_{i}$$

Whole reorganization entropy equals:

(42) $$H_{reorg}=\sum_{i}^{prod}\omega_{i}h_{i}-\sum_{j}^{react}\omega_{j}h_{j}$$

**Figure 12:**
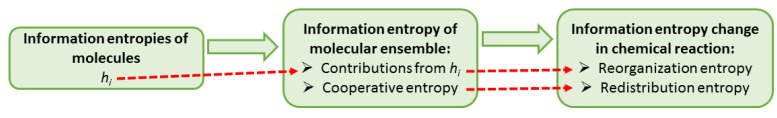
The origins of the quantities used for calculating information entropies of reactions according to [47].

These considerations allow treating a chemical reaction as a process of changes in the molecular structure and molecular size and quantifying the corresponding contributions (Figure 12) [47]. Note that the redistribution term depends only on ω*_i_* values as the reorganization terms depend on both ω*_i_* and *h_i_* values.

The information entropy of a chemical reaction can be also found without the consideration of the *h* values of the participants. As deduced in [46], it is completely attributed to appearing and disappearing atom types:

(43) $$\Delta h_{R}=-\sum_{j=1}^{n_{p}}p_{j}\log_{2}p_{j}-\sum_{j=1}^{n_{r}}r_{j}\log_{2}r_{j}$$

where *p* and *r* designate values corresponding to products and reactants.

**Figure 13:**
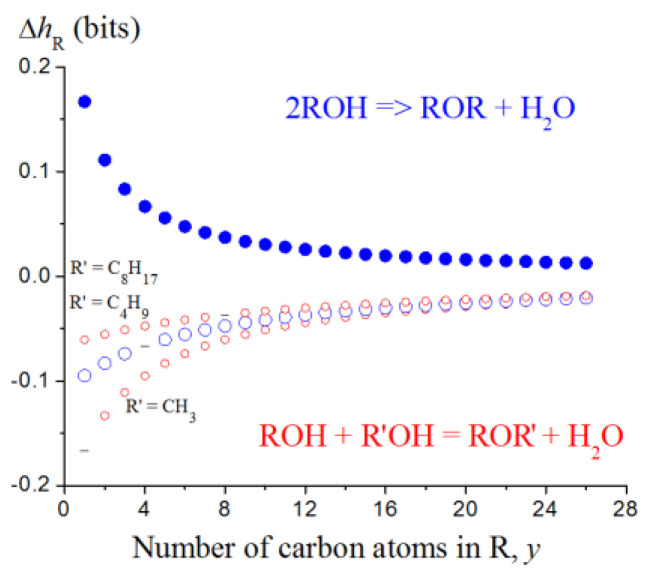
Information entropy of etherification. The points of symmetrical ethers ROR falling out from the dependencies for the formation of ROR’ are shown by dashes. They form their own trends shown with blue solid circles. Taken from [46] © 2018 Elsevier.

This approach has been tested on the limited number of chemical reactions but some general regularities have been obtained (Table 8). It provides the results consistent with common chemical notions. Additionally, it allows for digitally discriminating the subclasses of reactions depending on the type of reacting species. This is clearly demonstrated with the etherification reaction. The sign of the Δ*h*_R_ effect and plots *h* vs. the length of alkyl chains in ROH molecules differ depending on whether identical or different alcohol molecules react (Figure 13). In both cases (R = R’ and R ≠ R’), the absolute values of Δ*h*_R_ asymptotically approach to zero with the increase in the length of the carbon chain. It means that the same reaction in larger molecular ensembles induces smaller changes in the information entropy.

## 6. Discrete Information Entropy Approach and Some Aspects of Physical and Digital Chemistry

### 6.1. Everlasting Comparison of Information and Thermodynamic Entropies

Information and entropy are vague concepts of physics, and its vagueness has been inherited in chemistry. These are close concepts, as both of them are based on probability, and the relations between them have been considered almost since the birth of the quantitative information theory: e.g., in thinking experiments such as Szilard’s engine and Maxwell’s demon [114]. These theoretical models strongly justify the link between entropy and information and some scientists assumed that they are two antagonistic phenomena of one quantity (so-called the concept of entropy–information dualism). In addition to the closeness, we must mention the differences between information and entropy. For example, Kadomtsev stresses that their sameness based only on their probabilistic nature is just formal [115]. Trying to find the regularities, we should remember that the information entropy of the molecule characterizes its structural complexity, whereas the thermodynamic entropy is the function of matter approximated as the sum of translational, rotational, and vibrational contributions:

(44) $$S=S_{trans}+S_{rot}+S_{vib}$$

It means that *h* originates from one source, as the contributions to *S* are more diversified. Despite this, theoretical chemists try to find the correlations between information and thermodynamic entropies. For example, as found in [44], the information and thermodynamic entropies of some isomeric C_60_ fullerenes are symbatic. We think this is due to the structure of fullerenes: regular, rigid, and the same molecular size. Therefore, most fullerene isomers have almost equal *S*_trans_ and *S*_vib_ values and differ in *S*_rot_ ~ 1/σ. In Section 3.1, we indicated rotational symmetry number σ as the factor reducing the information entropy, hence we can roughly assume *h* ~ 1/σ. In extended fullerene series (e.g., the C_84_ isomers [59]), there is no overall *S* vs. *h* correlation. The correlations between the entropies of atomization and information entropies of saturated hydrocarbons reported in [46] are not of a great importance because both quantities strongly depend on molecular size.

Interesting results have been obtained for information entropy of mixing molecules, which form a molecular ensemble [46,47]:

(45) $$\Delta h_{mix}=h_{\mathrm{ME}}-\sum_{i=1}^{m}h_{i}=H_{\Omega }-\sum_{i=1}^{m}\left(1-\omega_{i}\right)h_{i}$$

It can be negative (e.g., when mixing molecules are the same), positive (when mixing zero-*h* and nonzero-*h* molecules), or zero. According to Equation (44), there are two antagonizing factors that define mixing: *H*_Ω_ increases Δ*h_mix_* whereas Σ(1 − *ω_i_*)*h_i_* decreases. The latter value becomes zero if *h_i_* = 0, which may be both when mixed molecules are the same (then *H*_Ω_ = 0 and Δ*h_mix_* = 0) or not (Δ*h_mix_* = *H*_Ω_). Otherwise, the sign of Δ*h_mix_* is a result of the balance between *H*_Ω_ and Σ(1 − *ω_i_*)*h_i_*.

We interpret [47] the information of mixing molecules in the aspect of the resources necessary to code the structures of initial molecules and resulting ensemble. For example, when *m* identical molecules separated, we need *h* bits per each molecule to describe the structures or *mh* in total. Their ensemble has *h*_ME_ = *h* (Equation (31)) that accounts the sameness of the molecules and reducing information resources to code. This is reflected by negative value Δ*h_mix_* = (1 – *m*)*h*.

In addition to mixing identical zero-*h* molecules, there is another case when Δ*h_mix_* = 0. For magic ensembles mentioned in Section 4.2:

(46) $$\Delta h_{mix}=\left(2-n\right)h$$

Hence, Δ*h_mix_* = 0 if the magic ensemble is bimolecular (*n* = 2). The examples of such ensembles are shown in Table 9. Thus, in contrast to thermodynamic entropy, the information entropy of mixing may obtain zero value when different species are mixed and, vice versa, the mixing of identical molecules having *h* ≠ 0 is characterized with Δ*h_mix_* ≠ 0.

### 6.2. Information Entropy and Physicochemical Processes

**Figure 14:**
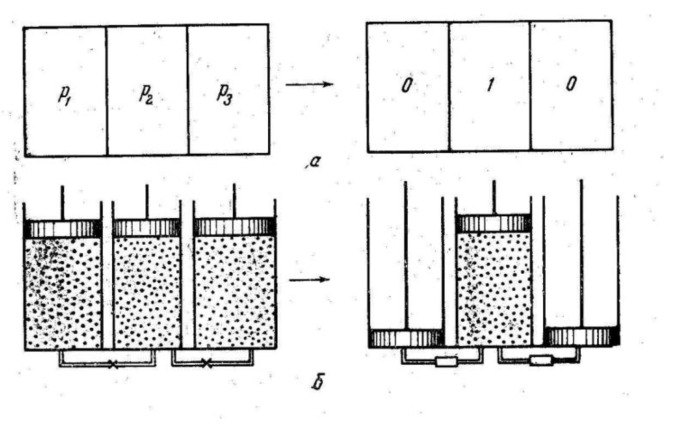
Kobozev’s thermodynamic model of reducing the information entropy. Explanations are in the text. Adapted from [116].

Information entropy estimates were used for the analysis of physical thermodynamic processes in early works reviewed in [114]. Numerous thinking experiments have been used to obtain the relations between information entropy and thermodynamic functions. We discuss one such approach, which has not been known to a wide audience. It is based on works [116,117] of Kobozev, who introduced a mechanistic thermodynamic model of the information process (Figure 14). It is a system of cylinders filled with ideal gas, with particles evenly distributed over them. When the connecting tubes are closed, the system is characterized with the set of probabilities *p_i_* ≠ 0 to find the portion of the gas in a certain cylinder. According to Equation (1), the information entropy of the system is non-zero.

Moving the pistons after the connecting tubes open, we collect all particles in one cylinder, hence *p_i_* = 0 except the filled one with *p* = 1. Zero information entropy corresponds to the system in the second state. Reducing information entropy creates the information, and this process requires the work applied to the system, which is called the work of information [116]. We clothe Kobozev’s formulations with the modern designations:

(47) $$A=\gamma \left(h_{2}-h_{1}\right)$$

where *h*_2_ and *h*_1_ are the information entropies of the system in the final and initial states and *γ* is the conversion coefficient between bits and energy units. Note that in [116], the information entropy is considered as the uncertainty and even the state with *h* = 0 relates not to the actual fact but to its possibility.

Though the formulations of [116,117] are quite rough, the idea to connect the information and energy parameters is very attractive in the aspect of structural chemistry. Indeed, if we assume that the *h* values of Equation (47) relate to the descriptors of chemical structure instead of thermodynamic probabilities, we will be able to assess the energy (the work) required for converting structure with *h*_1_ to structure with *h*_2_. These relations have never been explored but seem useful for chemical algebra approaches and reaction informatics [118], which aimed to find out the synthetic paths to a target compound with optimal energy cost and resources. This is also important in the aspect of the information entropy of chemical reactions as it may open opportunities to attribute energy changes to the contributions to the information entropy of reaction (and separately estimate redistribution and reorganization entropies).

### 6.3. Information Entropy and Digital Chemistry

The use of information as a structural parameter is also interpreted in terms of information processing. Information entropy *h* of the molecule calculated with Equation (9) provides the lower bound of the resources required for coding the structure of the molecule. This minimum implies distinguishing the inequivalent atoms in the chemical system.

The *h* values could be seen from the other side. They establish the upper bound of the information, which can be coded by the considered molecule. The latter means that all its atoms participate in recording. Of course, we neglect the questions about the stability of such information, its reading, and advisability in this interpretation.

Additional advances are expected from the information estimates of imperfect structures. In dendrimer series, we have found that the stable compounds with closed shells have lower information entropies than the intermediate structures with incompletely filled external shells [102]. Therefore, we assume that the information capacity must be temporarily increased to move the system from one informationally stable state to another. This assumption is reminiscent to the concept of the transition state of chemical reaction: its energy is higher than the energies of the reactants and products, and the reaction is associated with overcoming this activation barrier. In our case, there is a similar ‘information–entropy barrier’ required for switching the system between the informationally stable states.

At the end of this chapter, we pay attention to the fact that Equation (31) obtained for information entropy of molecular ensemble [47] is similar to the definition of the von Neumann entropy of several mixed states [15] used in quantum information processing. This suggests that there may be other similarities between ‘pure’ chemical objects and quantum information.

## 7. Applying Information Entropy to Nucleic Acids

At the end of the review, we briefly note that one of the first molecules analyzed in terms of the title approach were nucleic acids, biopolymers that code genomic information. However, the application of information entropy and related quantities to DNA and RNA molecules is a separate story [119,120,121]. We do not discuss it in the review because this case is far from chemical structure issues and closer to biological information. Of course, the functioning of natural information-bearing biomolecules is based on their chemical properties, but there is a hypothetical possibility of replacing them with other chemical substances (similar or not). In this sense, biochemical and biological information is macroscopic rather than microscopic. This makes applications of information entropy to DNA/RNA very similar to its applications to the text analysis: nucleotides play the role of letters, which compose the words—sentences. Consequently, the tasks solved with the information entropy differ from typical chemical ones: assessing the role of mutations [122], comparing random and regular sequences [123], discriminating DNAs of different types [124], evaluating the efficiency of natural genome codification [125], etc. Of course, there are works with chemical relevance (e.g., comparing thermodynamic stability and coding efficiency of DNA [126]), but they are currently scarce.

## 8. Conclusions

Information entropy is one of the concepts deeply rooted in modern chemistry. It is applied to estimate the complexity of molecules and molecular ensembles, the electronic structure of the molecules, signal processing, physicochemical processes, etc. Information entropy linked with chemical structure, thermodynamic entropy, energy, and computer sciences can take an important place between these fields and give a start for novel interdisciplinary studies.

There is a disadvantage of the information entropy approach due to its ignoring of the semantic aspects of information. It could be overcome in chemistry through the following modes:

- in terms of novel theories of semantic information;
- using information entropy in combination with other structural properties (molecular size, oxidation state, etc.);
- limiting the considered semantic field (for example, applying information entropy only to the limited isomeric or homologue series, reactions of one type, etc.).

## Figures and Tables

**Table 1:** Information entropies of *N*-atomic species. Taken from [47] © 2020 Elsevier.

Partition	*h* (Bits)	Examples
Diatomic species		
1 × 2	0	All homonuclear diatomic species A_2_ (e.g., H_2_, H_2_^+^, and O_2_)
2×1	1	All heteronuclear diatomic species AB (e.g., HF, HD, and HO^•^)
Triatomic species		
1 × 3	0	Cyclic species A_3_ (e.g., hypothetical cyclic ozone O_3_)
1 × 2 + 1 × 1	0.918	Linear/angular species AAA (e.g., open ozone O_3_, N_3_^–^, and I_3_^–^) and angular ABA (e.g., H_2_O, H_2_S, and: CH_2_)
3 × 1	1.585	ABC (e.g., HCN, HNC, and HOD) and AAB (e.g., HOO^•^)
Tetraatomic species		
1 × 4	0	Tetrahedral A_4_ species (e.g., P_4_)
1 × 3 + 1 × 1	0.811	AB_3_ (e.g., ^•^CH_3_, NH_3_, PCl_3_, NO_3_^–^, and CO_3_^2–^)
2 × 2	1	ABBA (e.g., HC≡CH)
1 × 2 + 2 × 1	1.5	A_2_BC (e.g., H_2_C=O and H_2_C=S)
4 × 1	2	ABBC, ABCD, and ABBB (e.g., HC≡CCl, HCNO, and: C=C=C=O, respectively)

**Table 2:** Information entropies of typical molecules. Taken from [46] © 2018 Elsevier.

Molecule	Partition	*h* (Bits)
CH_4_	1 × 4 + 1 × 1	0.722
CH_3_Cl	1 × 3 + 2 × 1	1.371
C_2_H_6_	1 × 6 + 1 × 2	0.811
C_2_H_4_	1 × 4 + 1 × 2	0.918
C_2_H_2_	2 × 2	1.000
CH_3_OH	1 × 3 + 3 × 1	1.792
CH_3_CH_2_OH	1 × 3 + 1 × 2 + 4 × 1	2.419
CH_3_OCH_3_	1 × 6 + 1 × 2 + 1 × 1	1.224
CH_3_COOH	1 × 3 + 5 × 1	2.406
C_6_H_6_	2 × 6	1.000
C_60_ (*I_h_*)	1 × 60	0
C_70_ (*D*_5*h*_)	3 × 10 + 2 × 20	2.236

**Table 3:** Shannon aromaticity indices of typical aromatic hydrocarbons. The calculations with the density functional theory method B3LYP/6-31+G** performed in [75].

Molecule	Structural Formula	Shannon Aromaticity, SA × 10^–6^
Benzene	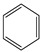	1.7 × 10^–6^
Naphthalene	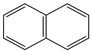	0.0737
Anthracene	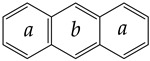	0.1378 (a) 0.0612 (b)
Phenanthrene	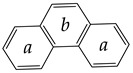	0.0042 (a) 0.1489 (b)

**Table 4:** Crystallographic point groups, abstract groups, and their complexity following from the partition of group elements into equivalence classes with respect to automorphisms of the group.

Crystal Class (Schoenflies Symbols)	Order	Partition of Group Elements	*h*, (Bits/Element)	*h_tot_*, (Bits/Group)
*C*_1_	1	{1}	0	0
*C*_2_, *C_i_*, *C_s_*	2	{1, 1}	1.000	2.000
*C*_3_	3	{1, 2}	0.918	2.755
*C*_4_, *S*_4_	4	{1, 1, 2}	1.500	6.000
*C*_6_, *S*_6_, *C*_3*h*_	6	{1, 1, 2, 2}	1.918	11.510
*C*_2*h*_, *C*_2*ʋ*_, *D*_2_	4	{1, 3}	0.811	3.245
*D*_2*h*_	8	{1, 7}	0.544	4.349
*C*_3*ʋ*_, *D*_2_	6	{1,2, 3}	1.459	8.755
*C*_4*h*_	8	{1, 1, 2, 4}	1.750	14.000
*C*_4*ʋ*_, *D*_4_, *D*_2*d*_	8	{1, 1, 2, 4}	1.750	14.000
*C*_6*h*_	12	{1, 2, 3, 6}	1.730	20.755
*C*_6*ʋ*_, *D*_6_, *D*_3*d*_, *D*_3*h*_	12	{1, 1, 2, 2, 6}	1.959	23.510
*D*_4*h*_	16	{1, 1, 2, 4, 8}	1.875	30.000
*D*_6*h*_	24	{1, 2, 3, 6, 12}	1.865	44.755
*T*	12	{1, 3, 8}	1.189	14.265
*T_h_*	24	{1, 1, 6, 8, 8}	1.939	46.529
*T_d_*, *O*	24	{1, 3, 6, 6, 8}	2.094	50.265
*O_h_*	48	{1, 1, 3, 8, 8, 12, 15}	2.369	113.700

**Table 5:** Classification of minerals based on their complexity according to [91].

Category	Total Information Content (Bits/Unit Cell)	Approximate Number of Mineral Species	Examples
Very simple	0–20	600	diamond, copper, halite, galena, uraninite, fluorite, quartz, corundum, ringwoodite, calcite, dolomite, zircon, goethite
Simple	20–100	1100	alunite, jarosite, nepheline, kieserite, szomolnokite, kaolinite, olivine-group minerals, diopside, orthoclase, albite, biotite 1M
Intermediate	100–500	1800	enstatite, epidote, biotite 2M_1_, leucite, apatite, natrolite, tale 2M, pyrope, grossular, beryl, muscovite 2M_1_, staurolite, actinolite, holmquistite, coesite, tourmaline, analcime, boracite
Complex	500–1000	300	eudialyte, steenstrupine, coquimbite, sapphirine, alum, cymrite, aluminite
Very complex	>1000	100	vesuvianite, paulingite, bouazzerite, asheroftine-(Y), bementite, antigorite

**Table 6:** Molecular complexity of selected molecular crystals $\tilde{I}$ (bits/atom) and I (bits/molecule). Taken from [100].

Compound	Ideal Symmetry of a Molecule		Real Symmetry of a Molecule	
	$\tilde{I}$	I	$\tilde{I}$	I
I_2_	0	0	0	0
S_6_	0	0	0	0
α-S_8_	0	0	2.000	16.000
α-N_2_	0	0	0	0
β-P_4_	0	0	3.585	14.340
C_60_	0	0	1.522	91.320
Ice *I_h_*	0.918	2.754	2.252	6.756
Benzene	1.000	12.000	2.585	31.020
Naphthalene	2.281	41.058	3.170	57.060

**Table 7:** Information entropies of typical dimorphic ensembles. Taken from [47] © 2020 Elsevier.

Ensemble *a*A + *b*B	Partition	*h*_A_ (Bits)	*h*_B_ (Bits)	*ω_max_*(A)	$h_{ME}^{max}\;(\mathrm{Bits})$
*a*NH_3_ + *b*N_2_	1 × *a* + 1 × 3*a* + 1 × 2*b*	0.811	0	0.637	1.462
*a*C_60_ + *b*O_2_	1 × 60*a* + 1 × 2*b*	0	0	0.5	1
*a*C_60_ + *b*O_3_	1 × 60*a* + 1 × *b* + 1 × 2*b*	0	0.918	0.346	1.531
*a*C_70_ + *b*O_3_	2 × 20*a* + 3 × 10*a* + 1 × *b* + 1 × 2*b*	2.236	0.918	0.714	2.723
*a*C_6_H_6_ + *b*O_3_	2 × 6*a* + 1 × *b* + 1 × 2*b*	1	0.918	0.514	1.960
*a*C_60_ + *b*C_2_H_2_	1 × 60*a* + 1 × 2*b*	0	1	0.333	1.585
*a*C_70_ + *b*C_2_H_2_	2 × 20*a* + 3 × 10*a* + + 1 × 2*b*	2.236	1	0.702	2.747
*a*C_6_H_6_ + *b*C_2_H_2_	2 × 6*a* + 2 × 2*b*	1	1	0.5	2

**Table 8:** Information entropy parameters of typical chemical reactions. Taken from [47] © 2020 Elsevier.

Reaction	Formal Equation	$h_{ME}^{prod}$	$h_{ME}^{react}$	*H_reorg_*	*H_redistr_*
Dissociation	D → A + B + … + C	$\sum^{\text{​}}\omega_{i}h_{i}+H_{\Omega }^{prod}$	*h*_D_	$\sum^{\text{​}}\omega_{i}h_{i}-h_{D}$	$H_{\Omega }^{prod}$
Addition	A + B + … + C → D	*h*_D_	$\sum^{\text{​}}\omega_{i}h_{i}+H_{\Omega }^{react}$	$h_{D}-\sum^{\text{​}}\omega_{i}h_{i}$	$-H_{\Omega }^{react}$
Atomization	A*_a_*B*_b_*…*_c_*C → *a*A + *b*B + … + *c*C	$H_{\Omega }^{prod}$	*h*(A*_a_*B*_b_*…*_c_*C)	*–h*(A*_a_*B*_b_*…*_c_*C)	$H_{\Omega }^{prod}$
Isomerization	A → B	*h*_B_	*h*_A_	*h*_B_ − *h*_A_	0

**Table 9:** Information entropy parameters of ‘magic’ molecular ensembles characterized with zero information entropy of mixing. Taken from [47].

Number of Molecules in Molecular Ensemble (*n*)	*h* = *H*_Ω_ (Bits)	*h*_ME_ (Bits)	Δ*h_mix_* (bits)	Examples
2	0.918	1.836	0	O_3_ + C_2_H_4_; :CH_2_ + C_2_H_4_; H_2_O + C_2_H_4_
2	0.811	1.622	0	intermetallic phases AB_3_ + A_3_B_9_
2	1	2	0	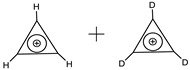
2	1	2	0